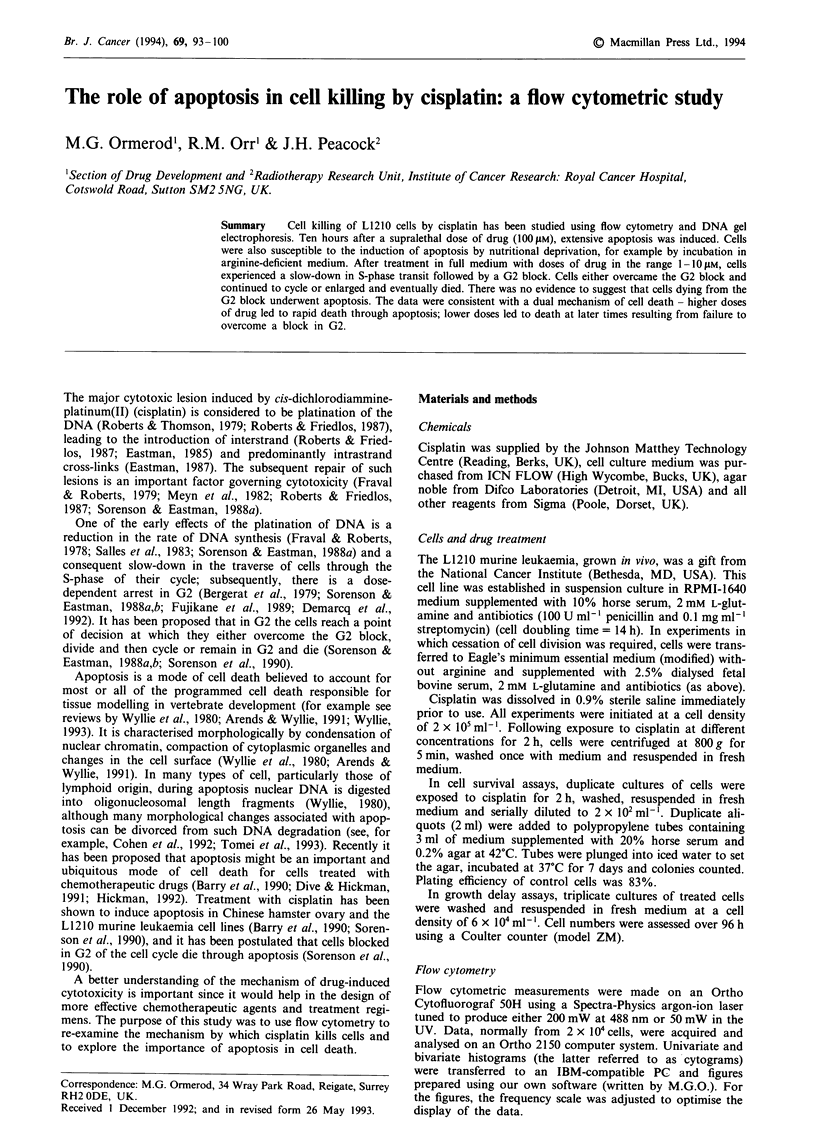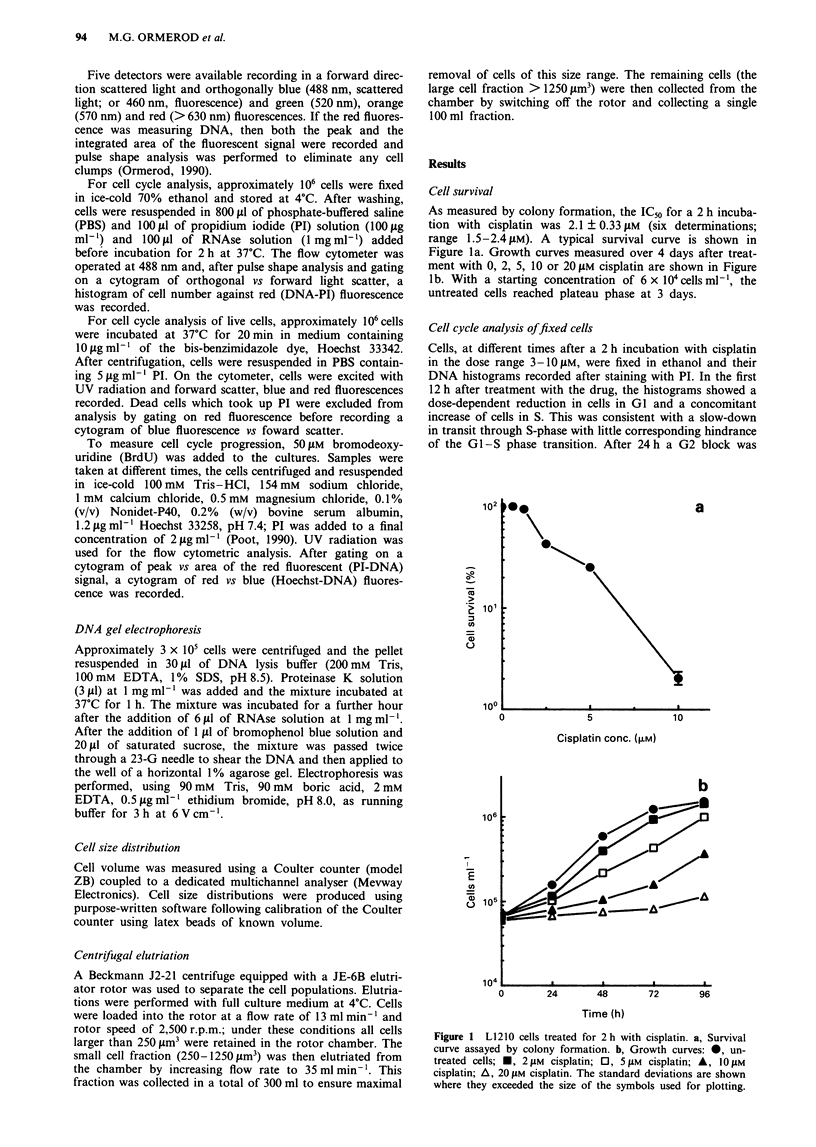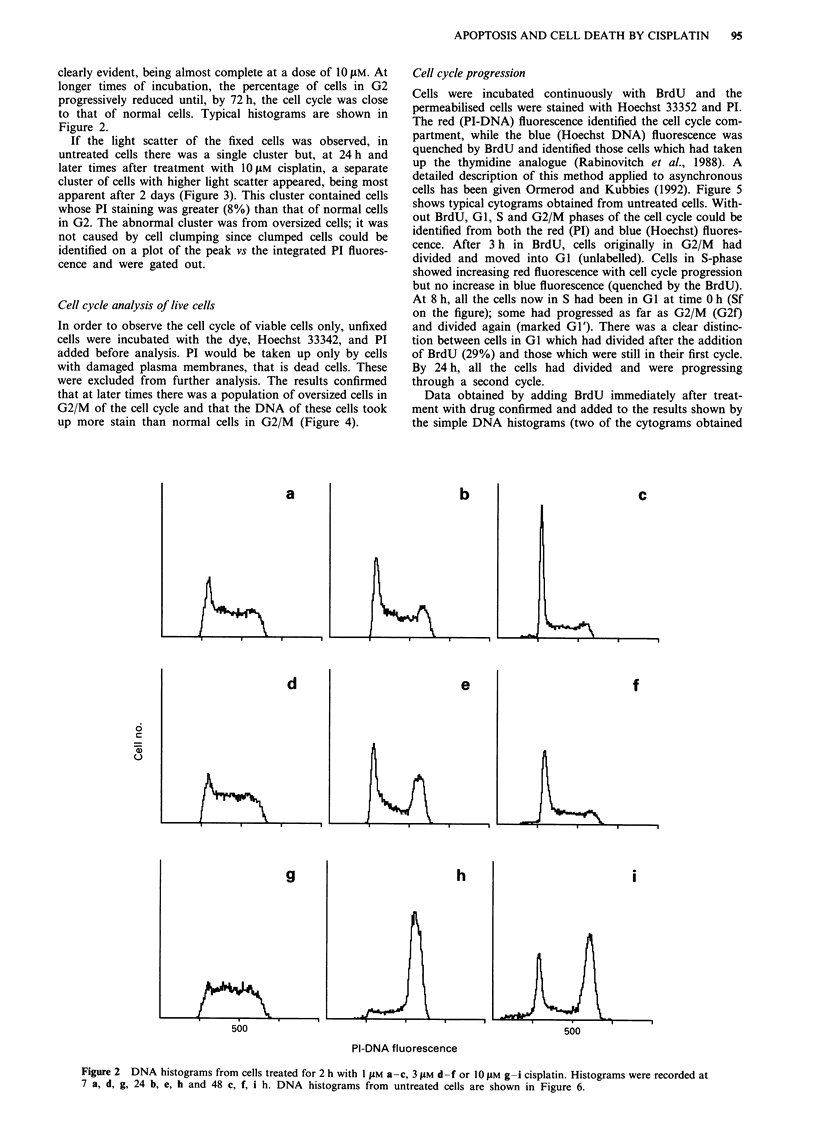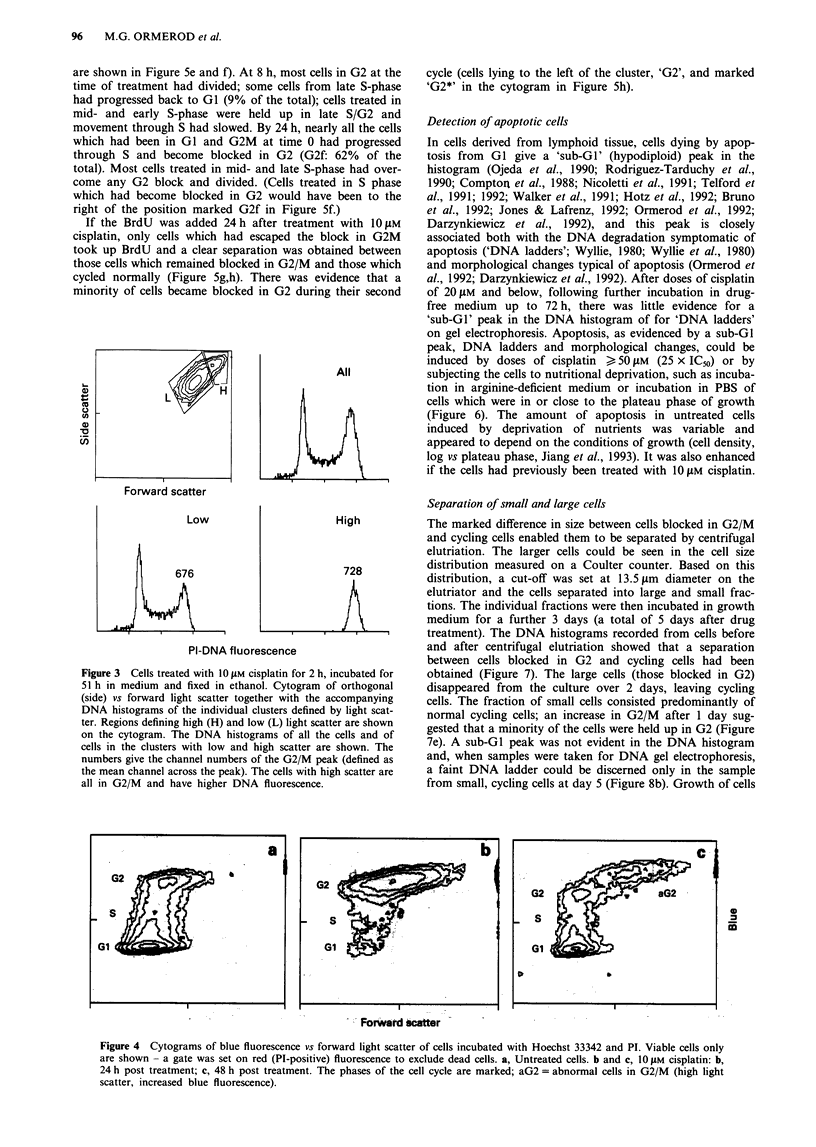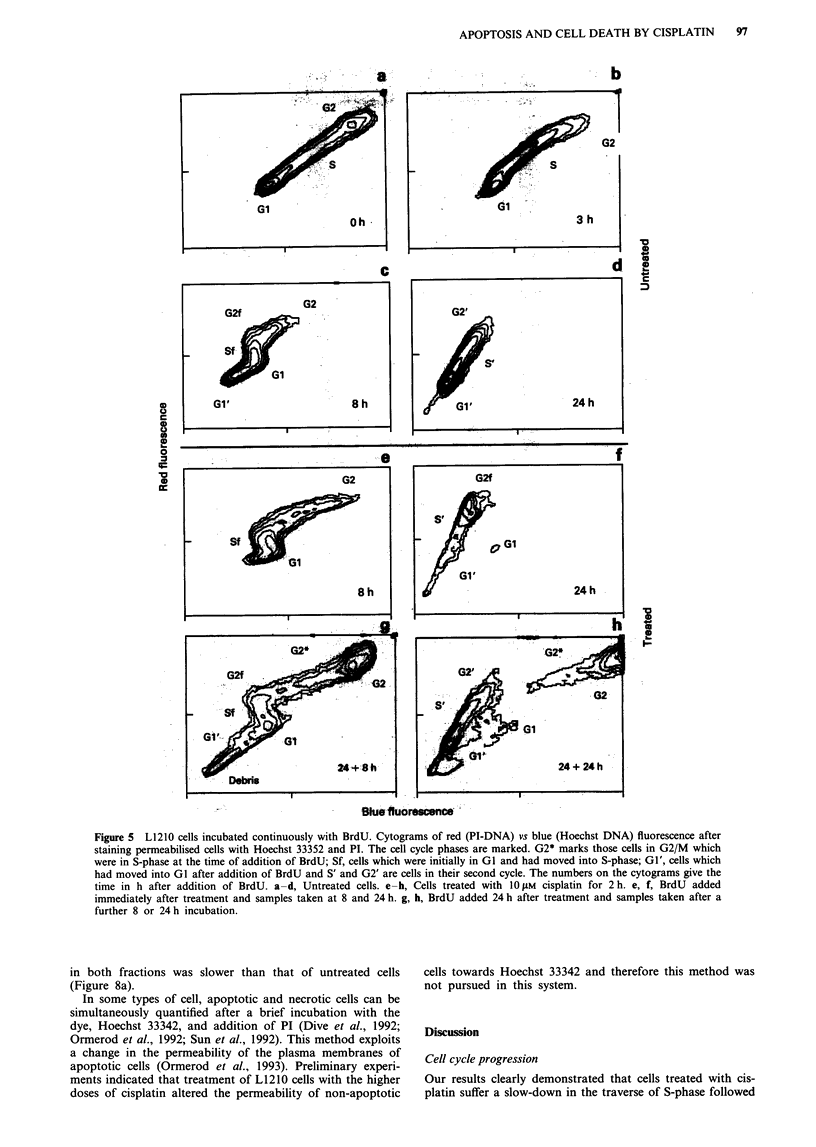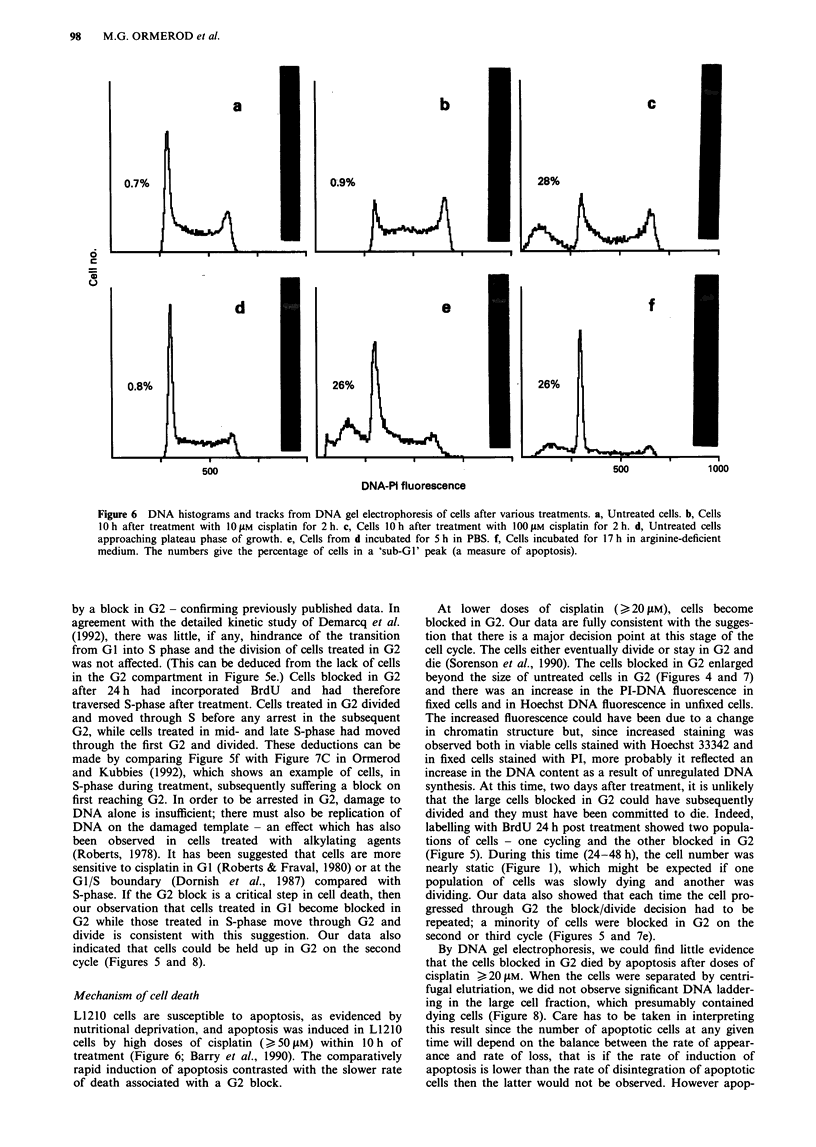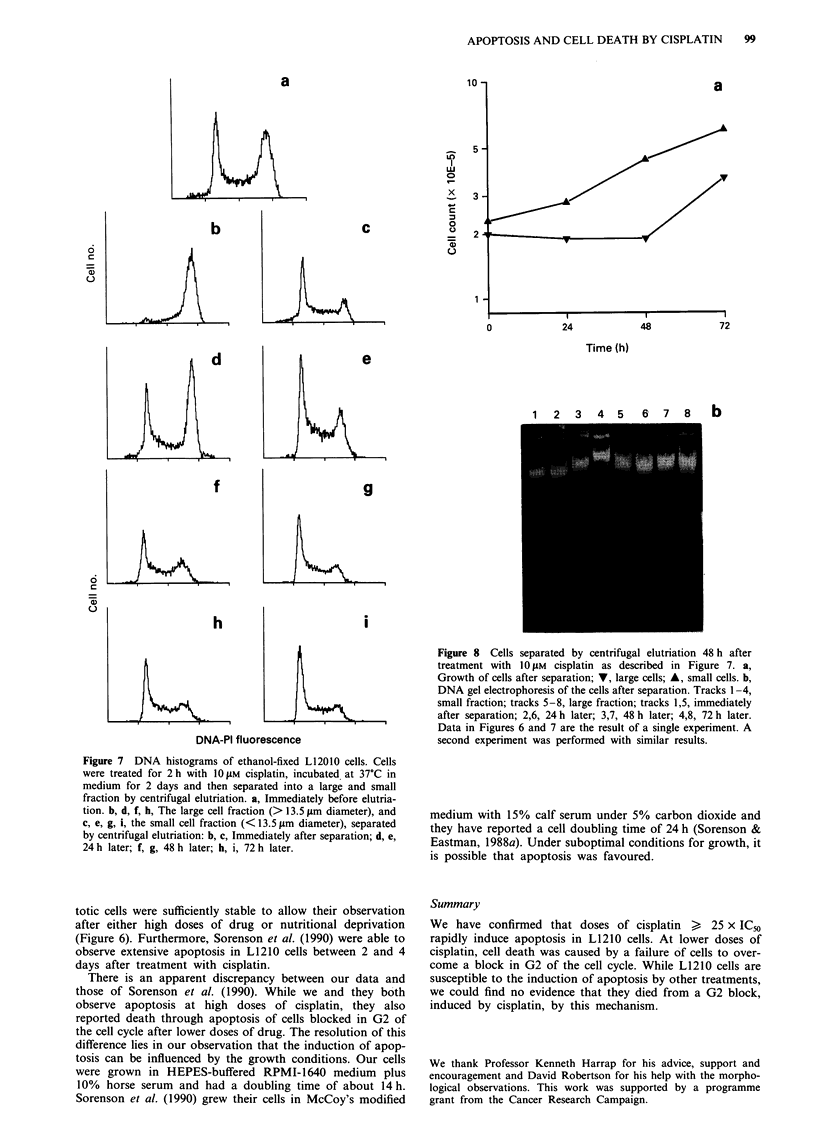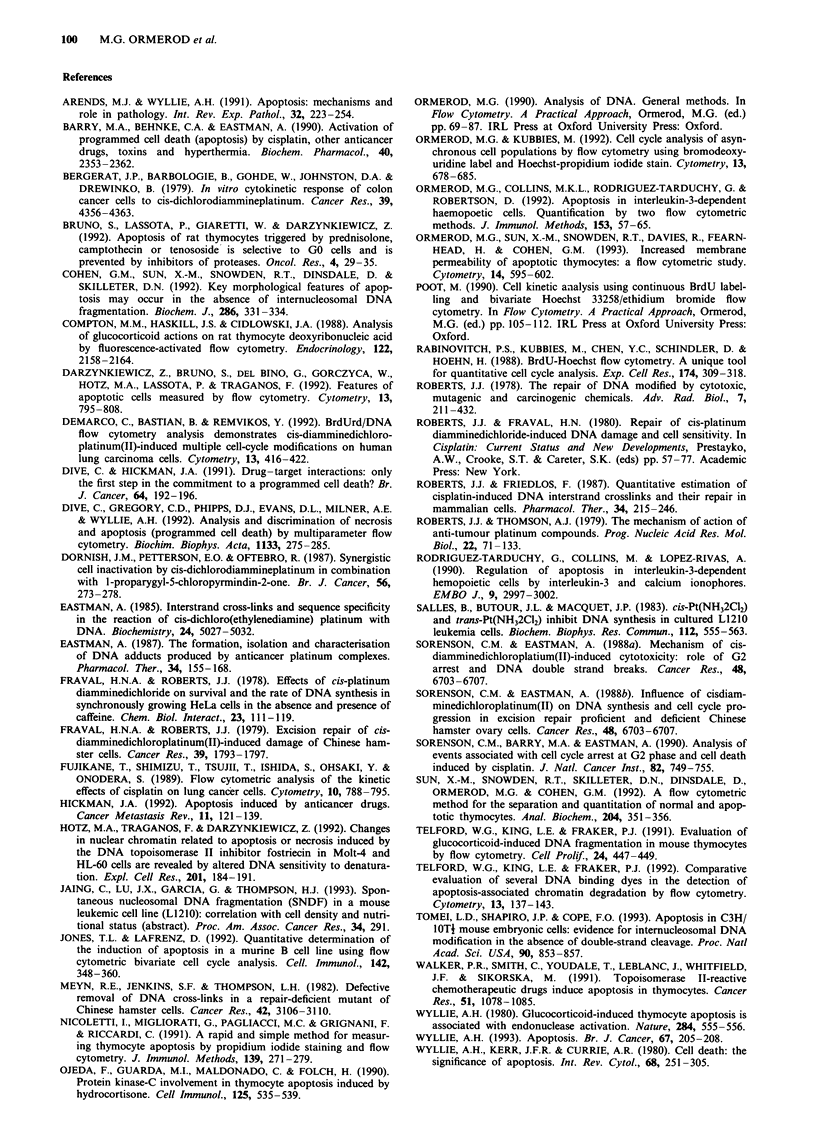# The role of apoptosis in cell killing by cisplatin: a flow cytometric study.

**DOI:** 10.1038/bjc.1994.14

**Published:** 1994-01

**Authors:** M. G. Ormerod, R. M. Orr, J. H. Peacock

**Affiliations:** Section of Drug Development, Institute of Cancer Research, Royal Cancer Hospital, Sutton, UK.

## Abstract

**Images:**


Br. J. Cancer (1994), 69, 93-100 © Macmillan Press Ltd., 1994

### The role of apoptosis in cell killing by cisplatin: a flow cytometric study

M.G. Ormerod[1], R.M. Orr[1] & J.H. Peacock[2]

[1]*Section of Drug Development and* [2]*Radiotherapy Research Unit, Institute of Cancer Research: Royal Cancer Hospital, Cotswold Road, Sutton SM2 5NG, UK.*

**Summary** Cell killing of L1210 cells by cisplatin has been studied using flow cytometry and DNA gel electrophoresis. Ten hours after a supralethal dose of drug (100 μM), extensive apoptosis was induced. Cells were also susceptible to the induction of apoptosis by nutritional deprivation, for example by incubation in arginine-deficient medium. After treatment in full medium with doses of drug in the range 1-10 μM, cells experienced a slow-down in S-phase transit followed by a G2 block. Cells either overcame the G2 block and continued to cycle or enlarged and eventually died. There was no evidence to suggest that cells dying from the G2 block underwent apoptosis. The data were consistent with a dual mechanism of cell death - higher doses of drug led to rapid death through apoptosis; lower doses led to death at later times resulting from failure to overcome a block in G2.

The major cytotoxic lesion induced by *cis*-dichlorodiammine-platinum(II) (cisplatin) is considered to be platination of the DNA (Roberts & Thomson, 1979; Roberts & Friedlos, 1987), leading to the introduction of interstrand (Roberts & Friedlos, 1987; Eastman, 1985) and predominantly intrastrand cross-links (Eastman, 1987). The subsequent repair of such lesions is an important factor governing cytotoxicity (Fraval & Roberts, 1979; Meyn *et al.*, 1982; Roberts & Friedlos, 1987; Sorenson & Eastman, 1988*a*).

One of the early effects of the platination of DNA is a reduction in the rate of DNA synthesis (Fraval & Roberts, 1978; Salles *et al.*, 1983; Sorenson & Eastman, 1988*a*) and a consequent slow-down in the traverse of cells through the S-phase of their cycle; subsequently, there is a dose-dependent arrest in G2 (Bergerat *et al.*, 1979; Sorenson & Eastman, 1988*a,b*; Fujikane *et al.*, 1989; Demarcq *et al.*, 1992). It has been proposed that in G2 the cells reach a point of decision at which they either overcome the G2 block, divide and then cycle or remain in G2 and die (Sorenson & Eastman, 1988*a,b*; Sorenson *et al.*, 1990).

Apoptosis is a mode of cell death believed to account for most or all of the programmed cell death responsible for tissue modelling in vertebrate development (for example see reviews by Wyllie *et al.*, 1980; Arends & Wyllie, 1991; Wyllie, 1993). It is characterised morphologically by condensation of nuclear chromatin, compaction of cytoplasmic organelles and changes in the cell surface (Wyllie *et al.*, 1980; Arends & Wyllie, 1991). In many types of cell, particularly those of lymphoid origin, during apoptosis nuclear DNA is digested into oligonucleosomal length fragments (Wyllie, 1980), although many morphological changes associated with apoptosis can be divorced from such DNA degradation (see, for example, Cohen *et al.*, 1992; Tomei *et al.*, 1993). Recently it has been proposed that apoptosis might be an important and ubiquitous mode of cell death for cells treated with chemotherapeutic drugs (Barry *et al.*, 1990; Dive & Hickman, 1991; Hickman, 1992). Treatment with cisplatin has been shown to induce apoptosis in Chinese hamster ovary and the L1210 murine leukaemia cell lines (Barry *et al.*, 1990; Sorenson *et al.*, 1990), and it has been postulated that cells blocked in G2 of the cell cycle die through apoptosis (Sorenson *et al.*, 1990).

A better understanding of the mechanism of drug-induced cytotoxicity is important since it would help in the design of more effective chemotherapeutic agents and treatment regimens. The purpose of this study was to use flow cytometry to re-examine the mechanism by which cisplatin kills cells and to explore the importance of apoptosis in cell death.

Correspondence: M.G. Ormerod, 34 Wray Park Road, Reigate, Surrey RH2 0DE, UK.
Received 1 December 1992; and in revised form 26 May 1993.

### Materials and methods

### Chemicals

Cisplatin was supplied by the Johnson Matthey Technology Centre (Reading, Berks, UK), cell culture medium was purchased from ICN FLOW (High Wycombe, Bucks, UK), agar noble from Difco Laboratories (Detroit, MI, USA) and all other reagents from Sigma (Poole, Dorset, UK).

### Cells and drug treatment

The L1210 murine leukaemia, grown *in vivo*, was a gift from the National Cancer Institute (Bethesda, MD, USA). This cell line was established in suspension culture in RPMI-1640 medium supplemented with 10% horse serum, 2 mM L-glutamine and antibiotics (100 U $ml^{-1}$ penicillin and 0.1 mg $ml^{-1}$ streptomycin) (cell doubling time = 14 h). In experiments in which cessation of cell division was required, cells were transferred to Eagle's minimum essential medium (modified) without arginine and supplemented with 2.5% dialysed fetal bovine serum, 2 mM L-glutamine and antibiotics (as above).

Cisplatin was dissolved in 0.9% sterile saline immediately prior to use. All experiments were initiated at a cell density of $2 \times 10^5$ $ml^{-1}$. Following exposure to cisplatin at different concentrations for 2 h, cells were centrifuged at 800 *g* for 5 min, washed once with medium and resuspended in fresh medium.

In cell survival assays, duplicate cultures of cells were exposed to cisplatin for 2 h, washed, resuspended in fresh medium and serially diluted to $2 \times 10^2$ $ml^{-1}$. Duplicate aliquots (2 ml) were added to polypropylene tubes containing 3 ml of medium supplemented with 20% horse serum and 0.2% agar at 42°C. Tubes were plunged into iced water to set the agar, incubated at 37°C for 7 days and colonies counted. Plating efficiency of control cells was 83%.

In growth delay assays, triplicate cultures of treated cells were washed and resuspended in fresh medium at a cell density of $6 \times 10^4$ $ml^{-1}$. Cell numbers were assessed over 96 h using a Coulter counter (model ZM).

### Flow cytometry

Flow cytometric measurements were made on an Ortho Cytofluorograf 50H using a Spectra-Physics argon-ion laser tuned to produce either 200 mW at 488 nm or 50 mW in the UV. Data, normally from $2 \times 10^4$ cells, were acquired and analysed on an Ortho 2150 computer system. Univariate and bivariate histograms (the latter referred to as cytograms) were transferred to an IBM-compatible PC and figures prepared using our own software (written by M.G.O.). For the figures, the frequency scale was adjusted to optimise the display of the data.

Five detectors were available recording in a forward direction scattered light and orthogonally blue (488 nm, scattered light; or 460 nm, fluorescence) and green (520 nm), orange (570 nm) and red (> 630 nm) fluorescences. If the red fluorescence was measuring DNA, then both the peak and the integrated area of the fluorescent signal were recorded and pulse shape analysis was performed to eliminate any cell clumps (Ormerod, 1990).

For cell cycle analysis, approximately $10^6$ cells were fixed in ice-cold 70% ethanol and stored at 4°C. After washing, cells were resuspended in 800 µl of phosphate-buffered saline (PBS) and 100 µl of propidium iodide (PI) solution (100 µg $ml^{-1}$) and 100 µl of RNAse solution (1 mg $ml^{-1}$) added before incubation for 2 h at 37°C. The flow cytometer was operated at 488 nm and, after pulse shape analysis and gating on a cytogram of orthogonal *vs* forward light scatter, a histogram of cell number against red (DNA-PI) fluorescence was recorded.

For cell cycle analysis of live cells, approximately $10^6$ cells were incubated at 37°C for 20 min in medium containing 10 µg $ml^{-1}$ of the bis-benzimidazole dye, Hoechst 33342. After centrifugation, cells were resuspended in PBS containing 5 µg $ml^{-1}$ PI. On the cytometer, cells were excited with UV radiation and forward scatter, blue and red fluorescences recorded. Dead cells which took up PI were excluded from analysis by gating on red fluorescence before recording a cytogram of blue fluorescence *vs* foward scatter.

To measure cell cycle progression, 50 µM bromodeoxyuridine (BrdU) was added to the cultures. Samples were taken at different times, the cells centrifuged and resuspended in ice-cold 100 mM Tris-HCl, 154 mM sodium chloride, 1 mM calcium chloride, 0.5 mM magnesium chloride, 0.1% (v/v) Nonidet-P40, 0.2% (w/v) bovine serum albumin, 1.2 µg $ml^{-1}$ Hoechst 33258, pH 7.4; PI was added to a final concentration of 2 µg $ml^{-1}$ (Poot, 1990). UV radiation was used for the flow cytometric analysis. After gating on a cytogram of peak *vs* area of the red fluorescent (PI-DNA) signal, a cytogram of red *vs* blue (Hoechst-DNA) fluorescence was recorded.

### *DNA gel electrophoresis*

Approximately $3 \times 10^5$ cells were centrifuged and the pellet resuspended in 30 µl of DNA lysis buffer (200 mM Tris, 100 mM EDTA, 1% SDS, pH 8.5). Proteinase K solution (3 µl) at 1 mg $ml^{-1}$ was added and the mixture incubated at 37°C for 1 h. The mixture was incubated for a further hour after the addition of 6 µl of RNAse solution at 1 mg $ml^{-1}$. After the addition of 1 µl of bromophenol blue solution and 20 µl of saturated sucrose, the mixture was passed twice through a 23-G needle to shear the DNA and then applied to the well of a horizontal 1% agarose gel. Electrophoresis was performed, using 90 mM Tris, 90 mM boric acid, 2 mM EDTA, 0.5 µg $ml^{-1}$ ethidium bromide, pH 8.0, as running buffer for 3 h at 6 V $cm^{-1}$.

### *Cell size distribution*

Cell volume was measured using a Coulter counter (model ZB) coupled to a dedicated multichannel analyser (Mevway Electronics). Cell size distributions were produced using purpose-written software following calibration of the Coulter counter using latex beads of known volume.

### *Centrifugal elutriation*

A Beckmann J2-21 centrifuge equipped with a JE-6B elutriator rotor was used to separate the cell populations. Elutriations were performed with full culture medium at 4°C. Cells were loaded into the rotor at a flow rate of 13 ml $min^{-1}$ and rotor speed of 2,500 r.p.m.; under these conditions all cells larger than 250 $\mu m^3$ were retained in the rotor chamber. The small cell fraction (250–1250 $\mu m^3$) was then elutriated from the chamber by increasing flow rate to 35 ml $min^{-1}$. This fraction was collected in a total of 300 ml to ensure maximal removal of cells of this size range. The remaining cells (the large cell fraction > 1250 $\mu m^3$) were then collected from the chamber by switching off the rotor and collecting a single 100 ml fraction.

### Results

### *Cell survival*

As measured by colony formation, the $IC_{50}$ for a 2 h incubation with cisplatin was 2.1 ± 0.33 µM (six determinations; range 1.5–2.4 µM). A typical survival curve is shown in Figure 1a. Growth curves measured over 4 days after treatment with 0, 2, 5, 10 or 20 µM cisplatin are shown in Figure 1b. With a starting concentration of $6 \times 10^4$ cells $ml^{-1}$, the untreated cells reached plateau phase at 3 days.

### *Cell cycle analysis of fixed cells*

Cells, at different times after a 2 h incubation with cisplatin in the dose range 3–10 µM, were fixed in ethanol and their DNA histograms recorded after staining with PI. In the first 12 h after treatment with the drug, the histograms showed a dose-dependent reduction in cells in G1 and a concomitant increase of cells in S. This was consistent with a slow-down in transit through S-phase with little corresponding hindrance of the G1–S phase transition. After 24 h a G2 block was

**Figure 1** L1210 cells treated for 2 h with cisplatin. **a**, Survival curve assayed by colony formation. **b**, Growth curves: ●, untreated cells; ■, 2 µM cisplatin; □, 5 µM cisplatin; ▲, 10 µM cisplatin; △, 20 µM cisplatin. The standard deviations are shown where they exceeded the size of the symbols used for plotting.

clearly evident, being almost complete at a dose of 10 μM. At longer times of incubation, the percentage of cells in G2 progressively reduced until, by 72 h, the cell cycle was close to that of normal cells. Typical histograms are shown in Figure 2.

If the light scatter of the fixed cells was observed, in untreated cells there was a single cluster but, at 24 h and later times after treatment with 10 μM cisplatin, a separate cluster of cells with higher light scatter appeared, being most apparent after 2 days (Figure 3). This cluster contained cells whose PI staining was greater (8%) than that of normal cells in G2. The abnormal cluster was from oversized cells; it was not caused by cell clumping since clumped cells could be identified on a plot of the peak *vs* the integrated PI fluorescence and were gated out.

### *Cell cycle analysis of live cells*

In order to observe the cell cycle of viable cells only, unfixed cells were incubated with the dye, Hoechst 33342, and PI added before analysis. PI would be taken up only by cells with damaged plasma membranes, that is dead cells. These were excluded from further analysis. The results confirmed that at later times there was a population of oversized cells in G2/M of the cell cycle and that the DNA of these cells took up more stain than normal cells in G2/M (Figure 4).

### *Cell cycle progression*

Cells were incubated continuously with BrdU and the permeabilised cells were stained with Hoechst 33352 and PI. The red (PI-DNA) fluorescence identified the cell cycle compartment, while the blue (Hoechst DNA) fluorescence was quenched by BrdU and identified those cells which had taken up the thymidine analogue (Rabinovitch *et al.*, 1988). A detailed description of this method applied to asynchronous cells has been given Ormerod and Kubbies (1992). Figure 5 shows typical cytograms obtained from untreated cells. Without BrdU, G1, S and G2/M phases of the cell cycle could be identified from both the red (PI) and blue (Hoechst) fluorescence. After 3 h in BrdU, cells originally in G2/M had divided and moved into G1 (unlabelled). Cells in S-phase showed increasing red fluorescence with cell cycle progression but no increase in blue fluorescence (quenched by the BrdU). At 8 h, all the cells now in S had been in G1 at time 0 h (Sf on the figure); some had progressed as far as G2/M (G2f) and divided again (marked G1′). There was a clear distinction between cells in G1 which had divided after the addition of BrdU (29%) and those which were still in their first cycle. By 24 h, all the cells had divided and were progressing through a second cycle.

Data obtained by adding BrdU immediately after treatment with drug confirmed and added to the results shown by the simple DNA histograms (two of the cytograms obtained

**Figure 2** DNA histograms from cells treated for 2 h with 1 μM **a**–**c**, 3 μM **d**–**f** or 10 μM **g**–**i** cisplatin. Histograms were recorded at 7 **a**, **d**, **g**, 24 **b**, **e**, **h** and 48 **c**, **f**, **i** h. DNA histograms from untreated cells are shown in Figure 6.

are shown in Figure 5e and f). At 8 h, most cells in G2 at the time of treatment had divided; some cells from late S-phase had progressed back to G1 (9% of the total); cells treated in mid- and early S-phase were held up in late S/G2 and movement through S had slowed. By 24 h, nearly all the cells which had been in G1 and G2M at time 0 had progressed through S and become blocked in G2 (G2f: 62% of the total). Most cells treated in mid- and late S-phase had overcome any G2 block and divided. (Cells treated in S phase which had become blocked in G2 would have been to the right of the position marked G2f in Figure 5f.)

If the BrdU was added 24 h after treatment with 10 µM cisplatin, only cells which had escaped the block in G2M took up BrdU and a clear separation was obtained between those cells which remained blocked in G2/M and those which cycled normally (Figure 5g,h). There was evidence that a minority of cells became blocked in G2 during their second cycle (cells lying to the left of the cluster, 'G2', and marked 'G2*' in the cytogram in Figure 5h).

**Figure 3** Cells treated with 10 µM cisplatin for 2 h, incubated for 51 h in medium and fixed in ethanol. Cytogram of orthogonal (side) *vs* forward light scatter together with the accompanying DNA histograms of the individual clusters defined by light scatter. Regions defining high (H) and low (L) light scatter are shown on the cytogram. The DNA histograms of all the cells and of cells in the clusters with low and high scatter are shown. The numbers give the channel numbers of the G2/M peak (defined as the mean channel across the peak). The cells with high scatter are all in G2/M and have higher DNA fluorescence.

*Detection of apoptotic cells*

In cells derived from lymphoid tissue, cells dying by apoptosis from G1 give a 'sub-G1' (hypodiploid) peak in the histogram (Ojeda *et al.*, 1990; Rodriguez-Tarduchy *et al.*, 1990; Compton *et al.*, 1988; Nicoletti *et al.*, 1991; Telford *et al.*, 1991; 1992; Walker *et al.*, 1991; Hotz *et al.*, 1992; Bruno *et al.*, 1992; Jones & Lafrenz, 1992; Ormerod *et al.*, 1992; Darzynkiewicz *et al.*, 1992), and this peak is closely associated both with the DNA degradation symptomatic of apoptosis ('DNA ladders'; Wyllie, 1980; Wyllie *et al.*, 1980) and morphological changes typical of apoptosis (Ormerod *et al.*, 1992; Darzynkiewicz *et al.*, 1992). After doses of cisplatin of 20 µM and below, following further incubation in drug-free medium up to 72 h, there was little evidence for a 'sub-G1' peak in the DNA histogram of for 'DNA ladders' on gel electrophoresis. Apoptosis, as evidenced by a sub-G1 peak, DNA ladders and morphological changes, could be induced by doses of cisplatin ⩾50 µM (25 × $IC_{50}$) or by subjecting the cells to nutritional deprivation, such as incubation in arginine-deficient medium or incubation in PBS of cells which were in or close to the plateau phase of growth (Figure 6). The amount of apoptosis in untreated cells induced by deprivation of nutrients was variable and appeared to depend on the conditions of growth (cell density, log *vs* plateau phase, Jiang *et al.*, 1993). It was also enhanced if the cells had previously been treated with 10 µM cisplatin.

*Separation of small and large cells*

The marked difference in size between cells blocked in G2/M and cycling cells enabled them to be separated by centrifugal elutriation. The larger cells could be seen in the cell size distribution measured on a Coulter counter. Based on this distribution, a cut-off was set at 13.5 µm diameter on the elutriator and the cells separated into large and small fractions. The individual fractions were then incubated in growth medium for a further 3 days (a total of 5 days after drug treatment). The DNA histograms recorded from cells before and after centrifugal elutriation showed that a separation between cells blocked in G2 and cycling cells had been obtained (Figure 7). The large cells (those blocked in G2) disappeared from the culture over 2 days, leaving cycling cells. The fraction of small cells consisted predominantly of normal cycling cells; an increase in G2/M after 1 day suggested that a minority of the cells were held up in G2 (Figure 7e). A sub-G1 peak was not evident in the DNA histogram and, when samples were taken for DNA gel electrophoresis, a faint DNA ladder could be discerned only in the sample from small, cycling cells at day 5 (Figure 8b). Growth of cells

**Figure 4** Cytograms of blue fluorescence *vs* forward light scatter of cells incubated with Hoechst 33342 and PI. Viable cells only are shown – a gate was set on red (PI-positive) fluorescence to exclude dead cells. **a**, Untreated cells. **b** and **c**, 10 µM cisplatin: **b**, 24 h post treatment; **c**, 48 h post treatment. The phases of the cell cycle are marked; aG2 = abnormal cells in G2/M (high light scatter, increased blue fluorescence).

**Figure 5** L1210 cells incubated continuously with BrdU. Cytograms of red (PI-DNA) *vs* blue (Hoechst DNA) fluorescence after staining permeabilised cells with Hoechst 33352 and PI. The cell cycle phases are marked. G2* marks those cells in G2/M which were in S-phase at the time of addition of BrdU; Sf, cells which were initially in G1 and had moved into S-phase; G1′, cells which had moved into G1 after addition of BrdU and S′ and G2′ are cells in their second cycle. The numbers on the cytograms give the time in h after addition of BrdU. **a**–**d**, Untreated cells. **e**–**h**, Cells treated with 10 μM cisplatin for 2 h. **e**, **f**, BrdU added immediately after treatment and samples taken at 8 and 24 h. **g**, **h**, BrdU added 24 h after treatment and samples taken after a further 8 or 24 h incubation.

in both fractions was slower than that of untreated cells (Figure 8a).

In some types of cell, apoptotic and necrotic cells can be simultaneously quantified after a brief incubation with the dye, Hoechst 33342, and addition of PI (Dive *et al.*, 1992; Ormerod *et al.*, 1992; Sun *et al.*, 1992). This method exploits a change in the permeability of the plasma membranes of apoptotic cells (Ormerod *et al.*, 1993). Preliminary experiments indicated that treatment of L1210 cells with the higher doses of cisplatin altered the permeability of non-apoptotic cells towards Hoechst 33342 and therefore this method was not pursued in this system.

### Discussion

### *Cell cycle progression*

Our results clearly demonstrated that cells treated with cisplatin suffer a slow-down in the traverse of S-phase followed

**Figure 6** DNA histograms and tracks from DNA gel electrophoresis of cells after various treatments. **a**, Untreated cells. **b**, Cells 10 h after treatment with 10 μM cisplatin for 2 h. **c**, Cells 10 h after treatment with 100 μM cisplatin for 2 h. **d**, Untreated cells approaching plateau phase of growth. **e**, Cells from **d** incubated for 5 h in PBS. **f**, Cells incubated for 17 h in arginine-deficient medium. The numbers give the percentage of cells in a 'sub-G1' peak (a measure of apoptosis).

by a block in G2 – confirming previously published data. In agreement with the detailed kinetic study of Demarcq *et al.* (1992), there was little, if any, hindrance of the transition from G1 into S phase and the division of cells treated in G2 was not affected. (This can be deduced from the lack of cells in the G2 compartment in Figure 5e.) Cells blocked in G2 after 24 h had incorporated BrdU and had therefore traversed S-phase after treatment. Cells treated in G2 divided and moved through S before any arrest in the subsequent G2, while cells treated in mid- and late S-phase had moved through the first G2 and divided. These deductions can be made by comparing Figure 5f with Figure 7C in Ormerod and Kubbies (1992), which shows an example of cells, in S-phase during treatment, subsequently suffering a block on first reaching G2. In order to be arrested in G2, damage to DNA alone is insufficient; there must also be replication of DNA on the damaged template – an effect which has also been observed in cells treated with alkylating agents (Roberts, 1978). It has been suggested that cells are more sensitive to cisplatin in G1 (Roberts & Fraval, 1980) or at the G1/S boundary (Dornish *et al.*, 1987) compared with S-phase. If the G2 block is a critical step in cell death, then our observation that cells treated in G1 become blocked in G2 while those treated in S-phase move through G2 and divide is consistent with this suggestion. Our data also indicated that cells could be held up in G2 on the second cycle (Figures 5 and 8).

### *Mechanism of cell death*

L1210 cells are susceptible to apoptosis, as evidenced by nutritional deprivation, and apoptosis was induced in L1210 cells by high doses of cisplatin (⩾50 μM) within 10 h of treatment (Figure 6; Barry *et al.*, 1990). The comparatively rapid induction of apoptosis contrasted with the slower rate of death associated with a G2 block.

At lower doses of cisplatin (⩾20 μM), cells become blocked in G2. Our data are fully consistent with the suggestion that there is a major decision point at this stage of the cell cycle. The cells either eventually divide or stay in G2 and die (Sorenson *et al.*, 1990). The cells blocked in G2 enlarged beyond the size of untreated cells in G2 (Figures 4 and 7) and there was an increase in the PI-DNA fluorescence in fixed cells and in Hoechst DNA fluorescence in unfixed cells. The increased fluorescence could have been due to a change in chromatin structure but, since increased staining was observed both in viable cells stained with Hoechst 33342 and in fixed cells stained with PI, more probably it reflected an increase in the DNA content as a result of unregulated DNA synthesis. At this time, two days after treatment, it is unlikely that the large cells blocked in G2 could have subsequently divided and they must have been committed to die. Indeed, labelling with BrdU 24 h post treatment showed two populations of cells – one cycling and the other blocked in G2 (Figure 5). During this time (24–48 h), the cell number was nearly static (Figure 1), which might be expected if one population of cells was slowly dying and another was dividing. Our data also showed that each time the cell progressed through G2 the block/divide decision had to be repeated; a minority of cells were blocked in G2 on the second or third cycle (Figures 5 and 7e).

By DNA gel electrophoresis, we could find little evidence that the cells blocked in G2 died by apoptosis after doses of cisplatin ⩾20 μM. When the cells were separated by centrifugal elutriation, we did not observe significant DNA laddering in the large cell fraction, which presumably contained dying cells (Figure 8). Care has to be taken in interpreting this result since the number of apoptotic cells at any given time will depend on the balance between the rate of appearance and rate of loss, that is if the rate of induction of apoptosis is lower than the rate of disintegration of apoptotic cells then the latter would not be observed. However apop-

**Figure 7** DNA histograms of ethanol-fixed L12010 cells. Cells were treated for 2 h with 10 μM cisplatin, incubated at 37°C in medium for 2 days and then separated into a large and small fraction by centrifugal elutriation. **a**, Immediately before elutriation. **b**, **d**, **f**, **h**, The large cell fraction (> 13.5 μm diameter), and **c**, **e**, **g**, **i**, the small cell fraction (< 13.5 μm diameter), separated by centrifugal elutriation: **b**, **c**, Immediately after separation; **d**, **e**, 24 h later; **f**, **g**, 48 h later; **h**, i, 72 h later.

**Figure 8** Cells separated by centrifugal elutriation 48 h after treatment with 10 μM cisplatin as described in Figure 7. **a**, Growth of cells after separation; ▼, large cells; ▲, small cells. **b**, DNA gel electrophoresis of the cells after separation. Tracks 1–4, small fraction; tracks 5–8, large fraction; tracks 1,5, immediately after separation; 2,6, 24 h later; 3,7, 48 h later; 4,8, 72 h later. Data in Figures 6 and 7 are the result of a single experiment. A second experiment was performed with similar results.

totic cells were sufficiently stable to allow their observation after either high doses of drug or nutritional deprivation (Figure 6). Furthermore, Sorenson *et al.* (1990) were able to observe extensive apoptosis in L1210 cells between 2 and 4 days after treatment with cisplatin.

There is an apparent discrepancy between our data and those of Sorenson *et al.* (1990). While we and they both observe apoptosis at high doses of cisplatin, they also reported death through apoptosis of cells blocked in G2 of the cell cycle after lower doses of drug. The resolution of this difference lies in our observation that the induction of apoptosis can be influenced by the growth conditions. Our cells were grown in HEPES-buffered RPMI-1640 medium plus 10% horse serum and had a doubling time of about 14 h. Sorenson *et al.* (1990) grew their cells in McCoy's modified medium with 15% calf serum under 5% carbon dioxide and they have reported a cell doubling time of 24 h (Sorenson & Eastman, 1988*a*). Under suboptimal conditions for growth, it is possible that apoptosis was favoured.

*Summary*

We have confirmed that doses of cisplatin ⩾ 25 × $IC_{50}$ rapidly induce apoptosis in L1210 cells. At lower doses of cisplatin, cell death was caused by a failure of cells to overcome a block in G2 of the cell cycle. While L1210 cells are susceptible to the induction of apoptosis by other treatments, we could find no evidence that they died from a G2 block, induced by cisplatin, by this mechanism.

We thank Professor Kenneth Harrap for his advice, support and encouragement and David Robertson for his help with the morphological observations. This work was supported by a programme grant from the Cancer Research Campaign.

### References

ARENDS, M.J. & WYLLIE, A.H. (1991). Apoptosis: mechanisms and role in pathology. *Int. Rev. Exp. Pathol.*, **32**, 223-254.

BARRY, M.A., BEHNKE, C.A. & EASTMAN, A. (1990). Activation of programmed cell death (apoptosis) by cisplatin, other anticancer drugs, toxins and hyperthermia. *Biochem. Pharmacol.*, **40**, 2353-2362.

BERGERAT, J.P., BARBOLOGIE, B., GOHDE, W., JOHNSTON, D.A. & DREWINKO, B. (1979). *In vitro* cytokinetic response of colon cancer cells to cis-dichlorodiammineplatinum. *Cancer Res.*, **39**, 4356-4363.

BRUNO, S., LASSOTA, P., GIARETTI, W. & DARZYNKIEWICZ, Z. (1992). Apoptosis of rat thymocytes triggered by prednisolone, camptothecin or tenososide is selective to G0 cells and is prevented by inhibitors of proteases. *Oncol. Res.*, **4**, 29-35.

COHEN, G.M., SUN, X.-M., SNOWDEN, R.T., DINSDALE, D. & SKILLETER, D.N. (1992). Key morphological features of apoptosis may occur in the absence of internucleosomal DNA fragmentation. *Biochem. J.*, **286**, 331-334.

COMPTON, M.M., HASKILL, J.S. & CIDLOWSKI, J.A. (1988). Analysis of glucocorticoid actions on rat thymocyte deoxyribonucleic acid by fluorescence-activated flow cytometry. *Endocrinology*, **122**, 2158-2164.

DARZYNKIEWICZ, Z., BRUNO, S., DEL BINO, G., GORCZYCA, W., HOTZ, M.A., LASSOTA, P. & TRAGANOS, F. (1992). Features of apoptotic cells measured by flow cytometry. *Cytometry*, **13**, 795-808.

DEMARCO, C., BASTIAN, B. & REMVIKOS, Y. (1992). BrdUrd/DNA flow cytometry analysis demonstrates cis-diamminedichloroplatinum(II)-induced multiple cell-cycle modifications on human lung carcinoma cells. *Cytometry*, **13**, 416-422.

DIVE, C. & HICKMAN, J.A. (1991). Drug-target interactions: only the first step in the commitment to a programmed cell death? *Br. J. Cancer*, **64**, 192-196.

DIVE, C., GREGORY, C.D., PHIPPS, D.J., EVANS, D.L., MILNER, A.E. & WYLLIE, A.H. (1992). Analysis and discrimination of necrosis and apoptosis (programmed cell death) by multiparameter flow cytometry. *Biochim. Biophys. Acta*, **1133**, 275-285.

DORNISH, J.M., PETTERSON, E.O. & OFTEBRO, R. (1987). Synergistic cell inactivation by cis-dichlorodiammineplatinum in combination with 1-proparygyl-5-chloropyrmindin-2-one. *Br. J. Cancer*, **56**, 273-278.

EASTMAN, A. (1985). Interstrand cross-links and sequence specificity in the reaction of cis-dichloro(ethylenediamine) platinum with DNA. *Biochemistry*, **24**, 5027-5032.

EASTMAN, A. (1987). The formation, isolation and characterisation of DNA adducts produced by anticancer platinum complexes. *Pharmacol. Ther.*, **34**, 155-168.

FRAVAL, H.N.A. & ROBERTS, J.J. (1978). Effects of *cis*-platinum diamminedichloride on survival and the rate of DNA synthesis in synchronously growing HeLa cells in the absence and presence of caffeine. *Chem. Biol. Interact.*, **23**, 111-119.

FRAVAL, H.N.A. & ROBERTS, J.J. (1979). Excision repair of *cis*-diamminedichloroplatinum(II)-induced damage of Chinese hamster cells. *Cancer Res.*, **39**, 1793-1797.

FUJIKANE, T., SHIMIZU, T., TSUJII, T., ISHIDA, S., OHSAKI, Y. & ONODERA, S. (1989). Flow cytometric analysis of the kinetic effects of cisplatin on lung cancer cells. *Cytometry*, **10**, 788-795.

HICKMAN, J.A. (1992). Apoptosis induced by anticancer drugs. *Cancer Metastasis Rev.*, **11**, 121-139.

HOTZ, M.A., TRAGANOS, F. & DARZYNKIEWICZ, Z. (1992). Changes in nuclear chromatin related to apoptosis or necrosis induced by the DNA topoisomerase II inhibitor fostriecin in Molt-4 and HL-60 cells are revealed by altered DNA sensitivity to denaturation. *Expl. Cell Res.*, **201**, 184-191.

JAING, C., LU, J.X., GARCIA, G. & THOMPSON, H.J. (1993). Spontaneous nucleosomal DNA fragmentation (SNDF) in a mouse leukemic cell line (L1210): correlation with cell density and nutritional status (abstract). *Proc. Am. Assoc. Cancer Res.*, **34**, 291.

JONES, T.L. & LAFRENZ, D. (1992). Quantitative determination of the induction of apoptosis in a murine B cell line using flow cytometric bivariate cell cycle analysis. *Cell. Immunol.*, **142**, 348-360.

MEYN, R.E., JENKINS, S.F. & THOMPSON, L.H. (1982). Defective removal of DNA cross-links in a repair-deficient mutant of Chinese hamster cells. *Cancer Res.*, **42**, 3106-3110.

NICOLETTI, I., MIGLIORATI, G., PAGLIACCI, M.C. & GRIGNANI, F. & RICCARDI, C. (1991). A rapid and simple method for measuring thymocyte apoptosis by propidium iodide staining and flow cytometry. *J. Immunol. Methods*, **139**, 271-279.

OJEDA, F., GUARDA, M.I., MALDONADO, C. & FOLCH, H. (1990). Protein kinase-C involvement in thymocyte apoptosis induced by hydrocortisone. *Cell Immunol.*, **125**, 535-539.

ORMEROD, M.G. (1990). Analysis of DNA. General methods. In *Flow Cytometry. A Practical Approach*, Ormerod, M.G. (ed.) pp. 69-87. IRL Press at Oxford University Press: Oxford.

ORMEROD, M.G. & KUBBIES, M. (1992). Cell cycle analysis of asynchronous cell populations by flow cytometry using bromodeoxyuridine label and Hoechst-propidium iodide stain. *Cytometry*, **13**, 678-685.

ORMEROD, M.G., COLLINS, M.K.L., RODRIGUEZ-TARDUCHY, G. & ROBERTSON, D. (1992). Apoptosis in interleukin-3-dependent haemopoetic cells. Quantification by two flow cytometric methods. *J. Immunol. Methods*, **153**, 57-65.

ORMEROD, M.G., SUN, X.-M., SNOWDEN, R.T., DAVIES, R., FEARNHEAD, H. & COHEN, G.M. (1993). Increased membrane permeability of apoptotic thymocytes: a flow cytometric study. *Cytometry*, **14**, 595-602.

POOT, M. (1990). Cell kinetic analysis using continuous BrdU labelling and bivariate Hoechst 33258/ethidium bromide flow cytometry. In *Flow Cytometry. A Practical Approach*, Ormerod, M.G. (ed.) pp. 105-112. IRL Press at Oxford University Press: Oxford.

RABINOVITCH, P.S., KUBBIES, M., CHEN, Y.C., SCHINDLER, D. & HOEHN, H. (1988). BrdU-Hoechst flow cytometry. A unique tool for quantitative cell cycle analysis. *Exp. Cell Res.*, **174**, 309-318.

ROBERTS, J.J. (1978). The repair of DNA modified by cytotoxic, mutagenic and carcinogenic chemicals. *Adv. Rad. Biol.*, **7**, 211-432.

ROBERTS, J.J. & FRAVAL, H.N. (1980). Repair of cis-platinum diamminedichloride-induced DNA damage and cell sensitivity. In *Cisplatin: Current Status and New Developments*, Prestayko, A.W., Crooke, S.T. & Careter, S.K. (eds) pp. 57-77. Academic Press: New York.

ROBERTS, J.J. & FRIEDLOS, F. (1987). Quantitative estimation of cisplatin-induced DNA interstrand crosslinks and their repair in mammalian cells. *Pharmacol. Ther.*, **34**, 215-246.

ROBERTS, J.J. & THOMSON, A.J. (1979). The mechanism of action of anti-tumour platinum compounds. *Prog. Nucleic Acid Res. Mol. Biol.*, **22**, 71-133.

RODRIGUEZ-TARDUCHY, G., COLLINS, M. & LOPEZ-RIVAS, A. (1990). Regulation of apoptosis in interleukin-3-dependent hemopoietic cells by interleukin-3 and calcium ionophores. *EMBO J.*, **9**, 2997-3002.

SALLES, B., BUTOUR, J.L. & MACQUET, J.P. (1983). *cis*-Pt($NH_3 2Cl_2$) and *trans*-Pt($NH_3 2Cl_2$) inhibit DNA synthesis in cultured L1210 leukemia cells. *Biochem. Biophys. Res. Commun.*, **112**, 555-563.

SORENSON, C.M. & EASTMAN, A. (1988*a*). Mechanism of cis-diamminedichloroplatium(II)-induced cytotoxicity: role of G2 arrest and DNA double strand breaks. *Cancer Res.*, **48**, 6703-6707.

SORENSON, C.M. & EASTMAN, A. (1988*b*). Influence of cisdiamminedichloroplatinum(II) on DNA synthesis and cell cycle progression in excision repair proficient and deficient Chinese hamster ovary cells. *Cancer Res.*, **48**, 6703-6707.

SORENSON, C.M., BARRY, M.A. & EASTMAN, A. (1990). Analysis of events associated with cell cycle arrest at G2 phase and cell death induced by cisplatin. *J. Natl. Cancer Inst.*, **82**, 749-755.

SUN, X.-M., SNOWDEN, R.T., SKILLETER, D.N., DINSDALE, D., ORMEROD, M.G. & COHEN, G.M. (1992). A flow cytometric method for the separation and quantitation of normal and apoptotic thymocytes. *Anal. Biochem.*, **204**, 351-356.

TELFORD, W.G., KING, L.E. & FRAKER, P.J. (1991). Evaluation of glucocorticoid-induced DNA fragmentation in mouse thymocytes by flow cytometry. *Cell Prolif.*, **24**, 447-449.

TELFORD, W.G., KING, L.E. & FRAKER, P.J. (1992). Comparative evaluation of several DNA binding dyes in the detection of apoptosis-associated chromatin degradation by flow cytometry. *Cytometry*, **13**, 137-143.

TOMEI, L.D., SHAPIRO, J.P. & COPE, F.O. (1993). Apoptosis in C3H/10T½ mouse embryonic cells: evidence for internucleosomal DNA modification in the absence of double-strand cleavage. *Proc. Natl Acad. Sci. USA*, **90**, 853-857.

WALKER, P.R., SMITH, C., YOUDALE, T., LEBLANC, J., WHITFIELD, J.F. & SIKORSKA, M. (1991). Topoisomerase II-reactive chemotherapeutic drugs induce apoptosis in thymocytes. *Cancer Res.*, **51**, 1078-1085.

WYLLIE, A.H. (1980). Glucocorticoid-induced thymocyte apoptosis is associated with endonuclease activation. *Nature*, **284**, 555-556.

WYLLIE, A.H. (1993). Apoptosis. *Br. J. Cancer*, **67**, 205-208.

WYLLIE, A.H., KERR, J.F.R. & CURRIE, A.R. (1980). Cell death: the significance of apoptosis. *Int. Rev. Cytol.*, **68**, 251-305.